# Association between work stress and mental health in Chinese public health workers during the COVID-19 epidemic: mediating role of social support and self-efficacy

**DOI:** 10.3389/fpubh.2023.1236645

**Published:** 2023-07-27

**Authors:** Yinqiao Dong, Qianqian Zhu, Ruijie Chang, Rongxi Wang, Yong Cai, Hong Huang

**Affiliations:** ^1^School of Public Health, Shanghai Jiao Tong University School of Medicine, Shanghai, China; ^2^Hongqiao International Institute of Medicine, Tongren Hospital, Shanghai Jiao Tong Univeristy School of Medicine, Shanghai, China

**Keywords:** depression, anxiety, social support, self-efficacy, healthcare worker, mediating effect

## Abstract

**Background:**

Little is known about the mediating mechanisms underlying the association between work stress and mental health, especially among primary public health workers (PHWs). We aimed to evaluated the association between work stress and mental health among PHWs, and explore the mediating roles of social support and self-efficacy.

**Methods:**

A large-scale cross-sectional survey was conducted among 3,809 PHWs from all 249 community health centers in 16 administrative districts throughout Shanghai, China. Pearson correlation and hierarchical linear regression were used to explore the associations among work stress, social support, self-efficacy and mental health. Structural equation modeling (SEM) was conducted to examine the mediation effects.

**Results:**

The prevalence of depression and anxiety symptoms among primary PHWs was 67.3 and 55.5%, respectively. There is a significant positive direct effect of work stress on mental health (β = 0.325, *p* < 0.001). Social support and self-efficacy partially mediated the relationship between work stress and mental health, respectively. Meanwhile, the chained mediating effects of social support and self-efficacy also buffered the predictive effects of work stress on anxiety and depression symptoms (β = 0.372, *p* < 0.001).

**Conclusion:**

Work stress has significant direct and indirect effects on mental health among primary PHWs. Enhancing social support and self-efficacy may be effective psychological interventions to mitigate the effects of work-related stress on mental health. These findings highlight the severity of mental health problems among primary public health workers and provide new evidence for early prevention and effective intervention strategies.

## Introduction

From early 2020 to the current time, the COVID-19 pandemic has affected many countries and regions and declared by the World Health Organization to be a public health emergency of international concern. Shanghai, one of the largest cities in Asia, experienced another unprecedented pandemic and associated lockdown in March 2022, which contributed to serious mental health problems (1). During the continuing COVID-19 pandemic, medical staff in hospitals and primary public health workers (PHWs) in community healthcare center (CHCs) faced increased workload and stress, resulting in adverse mental health conditions (2–4). Therefore, it is one of the main challenges of the pandemic to reduce the damage caused by COVID-19 to the mental health of healthcare workers. However, studies to date have focused on evaluating the mental health impact of COVID-19 related work stress on the various type of medical staff (5–7), with limited attention to primary public health workers. Furthermore, the neglect of primary public health workers may result in the accumulation of psychosocial problems caused by long-term, heavy work stress, potentially leading to a range of adverse psychosocial outcomes and harm to the primary healthcare system. Therefore, it is necessary to understand its harmful pathways and potential mechanisms on psychological health in order to take effective measures to prevent and reduce the risk of mental health problems, such as depression and anxiety symptoms, caused by work-related stress.

### Work stress during COVID-19 and mental health

Work stress on primary public health workers has increased mainly from the need that in addition to providing basic health services, public health physicians and medical technicians are required to perform routine nucleic acid testing, mass vaccinations, epidemiological investigations and surveillance, general practitioners and nurses are responsible for community fever clinic services, and administrators and other staff perform health promotion and education and other preventive and control measures to prevent the spread of COVID-19 in the community. Work stress can be quantified based on the classic theory of Effort-Reward Imbalance (ERI) which assesses the intense stress response induced by the imbalance between the effort and the reward of work (8). In the context of COVID-19, there is evidence that work stress is strongly associated with negative mental health outcomes among healthcare workers. Prolonged, intense work stress can directly contribute to the development of anxiety and depression disorders (9). Moreover, previous studies have confirmed that the beneficial predictive effect of ERI on increasing the risk of mental health problems and other adverse health outcomes (10, 11).

Mental health problems of PHWs in community, in addition to health care workers in hospital, is also a crucial part of public health event (12). An epidemiological survey of community epidemic prevention workers revealed that a considerable proportion of participants reported depression (39.7%) and anxiety (29.5%) symptoms (13). Anxiety and depression are common mental health problems (with high prevalence) among health care workers caused by the high-intensity work environment during the COVID-19 epidemic (2, 3, 14, 15). Although previous studies have provided preliminary evidence that work stress may be a significant predictor of mental health among medical staff or healthcare workers, there is limited research on the association between work stress and mental health problems among primary PHWs. Thus, there is a stronger need to further focus on mediating factors and explore potential pathways between work stress and depression and anxiety in order to provide effective interventions for reducing the mental health issues risks of primary PHWs.

### Social support and self-efficacy as mediators

Social support is a crucial interpersonal resource that encompasses mainly the close relationship between individuals and various aspects of society, such as friends, family and significant others. Based on stress buffering theory (16, 17), social support has a buffering effect on the relationship between work stress and mental health problems, which is also confirmed in healthcare workers (18, 19). Besides the external source and environment from social support, self-efficacy is an important internal aspect. Self-efficacy, which reflects individuals’ subjective evaluation of their own abilities, is considered an important personal trait with significant impact on coping with work stress and alleviating mental health problems (20, 21).

In addition, some studies have found that social support is an important source of self-efficacy, and the more social support someone receives, the more encouragement and affirmation they receive, which further enhances their self-efficacy (22–24). In contrast, when an individual perceives a lack of social support, this negative perspective on social relationships can lead to decrease in self-efficacy. This pathway proposed above bridges the gap between the external environment and personal factors. Notably, the mediating roles of social support and self-efficacy between job stress and mental health problems were also not confirmed in primary PHWs.

### Present study

Due to the limited epidemiological evidence for primary public health workers and the severity of public health challenges, we conducted a large-scale cross-sectional study of PHWs to explore the relationship between work stress, social support, self-efficacy, and mental health. Based on the theoretical model and previous related studies, we constructed a chain mediation model to confirm the following hypotheses: Hypothesis 1. Work stress can directly predict mental health. Hypothesis 2. Social support can mediate the association between work stress and mental health. Hypothesis 3. Self-efficacy can mediate the association between work stress and mental health. Hypothesis 4. Social support and self-efficacy are sequential mediators in the association between work stress and mental health (Supplementary Figure S1).

## Methods

### Participants

We performed a large-scale questionnaire survey among primary public health workers, covering all 249 community health service centers across all 16 districts throughout Shanghai. This study was conducted with the support of the Shanghai Municipal Health Commission and the cooperation of the leadership and administrative team of each CHC. The primary goal was to improve the capacity of primary care public health services and construction. From October to November 2022, this cross-sectional study was conducted *via* an online survey platform (“SurveyStar,” Changsha Ranxing Science and Technology, Shanghai, China). In order to ensure the accuracy and validity of the data, all questionnaires were set up in the computer system with intelligent logical checks to identify and reject invalid responses. The collected data were subsequently desensitized by specialists for statistical analysis. All respondents were invited to complete a self-assessment questionnaire through mobile phones, which included demographic information, lifestyle, work factors and psychological factors. A total of 3,937 respondents completed the questionnaire, and of whom 128 were excluded due to unwilling to engage in this survey. Finally, 3,809 valid questionnaires were collected, with the efficiency response rate of 96.75%. All participants were the target population and were informed of the significance and value of this anonymous survey before accessing the link to complete the questionnaire, and were then asked to read and sign an electronic informed consent form. This study was approved by the Ethical Review Committee of School of Public Health, Shanghai Jiao Tong University School of Medicine (approval number: SJUPN-202108) and adhered to the principles of the Declaration of Helsinki.

### Measures

#### General information

This survey contents included demographic variables (gender, age, educational level, marital status, hometown type), lifestyle characteristic (cigarette smoking, alcohol drinking, physical activity, sleep duration), occupation-related variables (type of occupation, professional title grade, years of working, length of public health service, daily working time, work overtime status, cumulative time involved in front-line prevention during the COVID-19 pandemic).

#### Work stress

Work stress was assessed by the Chinese version of the effort-reward imbalance (ERI) scale (25), which had good validity and reliability in Chinese medical staff (26, 27). The ERI scale contains 16-item of job effort (5 items, Cronbach’s α =0.90) and job reward (11 items, Cronbach’s α =0.82). All items were scored on a 5-point Likert scale ranging from 1 (strongly disagree) to 5 (strongly agree). ERI was measured by the effort-reward ratio calculated according to the formula: (effort total score) / [(reward total score) * (correction factor)], where the correction factor (5/11) considering the different number of items investigating job effort and reward (28). In this study, Cronbach’s alpha value of the ERI scale was 0.87.

#### Social support

The Multidimensional Scale of Perceived social support (MSPSS) was used to measure the levels of social support. It consists of three dimensions of support from family, friends, and others, with a total of 12 items. Each item of MSPSS is rated on a 7-point Likert scale ranging from one point (strongly disagree) to seven point (strongly agree). The total MSPSS score is based on the sum of three subscale ranging from 12 to 84, with higher scores representing higher levels of perceived social support (29). The reliability and validity of the Chinese version of the MSPSS have been demonstrated in different surveys (5, 30). In the present study, the Cronbach’s alpha was 0.98.

#### Self-efficacy

Generalized Self-Efficacy Scale (GSES) was developed to assess the level of self-efficacy through psychological states and behaviors that individuals might display when dealing with difficulties or setbacks. The Chinese version of this scale has been widely used among the Chinese population (31). This revised scale consists of 10 items, using 4-point Likert-type scale ranging from 1 (not at all true) and 4 (exactly true). The total scores range from 10 to 40, with higher scores reflecting a stronger sense of self-efficacy. Previous studies showed that the revised GSES scale has good reliability, validity (32). The Cronbach’s α in the current study was 0.94.

#### Anxiety

The General Anxiety Disorder-7 (GAD-7) is widely used to measure the severity of anxiety symptoms in the Chinese population (33). The GAD-7 consists of seven items, and each item is rated on 4-point Likert-type scales (0 = “not at all,” 1 = “a few days,” 2 = “more than half the days,” 3 = “almost every day”), summing to obtain a total score to measure the severity of anxiety symptoms. The higher the score, the more severe the anxiety. Participants with a total score of 0 to 4 were assessed as “normal mood,” 5 to 9 were assessed as “mild anxiety symptoms,” while scores above 10 or 15 represent moderate or severe anxiety, respectively (34). In this study, the Cronbach’s α of the scale was 0.97.

#### Depression

Depressive symptoms were assessed using the Patient Health Questionnaire-9 Items (PHQ-9). The PHQ-9 is an internationally used depressive symptom assessment scale that contains 9 items to assess the severity of depressive symptoms (35). The degree of depressive symptoms is based on the four answers ranging from 0 (not at all) to 3 (nearly every day), with the total score range from 0 to 27. Higher scores indicate higher levels of depression (0–4 = no depression, 5–9 = mild depression, 10–14 = moderate depression, 15–19 = severe depression, ≥20 = extremely severe depression) (36). Excellent validity and reliability for the PHQ-9 scale have been showed in Chinese hospital workers (37), and the current study showing a Cronbach α of 0.94 for depression.

### Statistical analysis

This study conducted descriptive analyses using frequencies and percentages for categorical variables and means ± standard deviations (SD) for continuous variables. Independent samples t-test and one-way ANOVA were used to compare differences in anxiety and depression scores across variable subgroups. The correlations between the main continuous variables (depression, anxiety, social support, self-efficacy, and ERI) were analyzed initially using the Pearson correlation method. Hierarchical multiple linear regression analysis was used to further explore the differential predictive effects of work stress on anxiety and depressive symptoms beyond social support and self-efficacy. The continuous anxiety and depression variables were considered as dependent variables, and demographic variables, lifestyle and COIVD-19-related and other work variables, work stress, social support and self-efficacy were controlled for in stepwise regression models 1–3, respectively. Before the mediation model analysis, the Harman’s single-factor test was conducted to examine the presence of common method bias in work stress, social support, self-efficacy, anxiety, and depression. It is generally considered that a variance of more than 40% for the first common factor indicated the presence of a common method bias (38, 39). Multiple mediation model analyses were conducted to examine the relationship between work stress, social support, self-efficacy, and mental health (depression and anxiety symptoms) using the maximum likelihood method. A bias-corrected bootstrap method (5,000 replicates) was applied to compute direct and indirect effects and 95% corrected confidence intervals (40). All statistical analyses were conducted using Mplus (version 8.4) and SPSS software (version 25.0). A two-tailed value of *p* < 0.05 was considered statistically significant.

## Results

### Characteristic of participants

**Figure 1:**
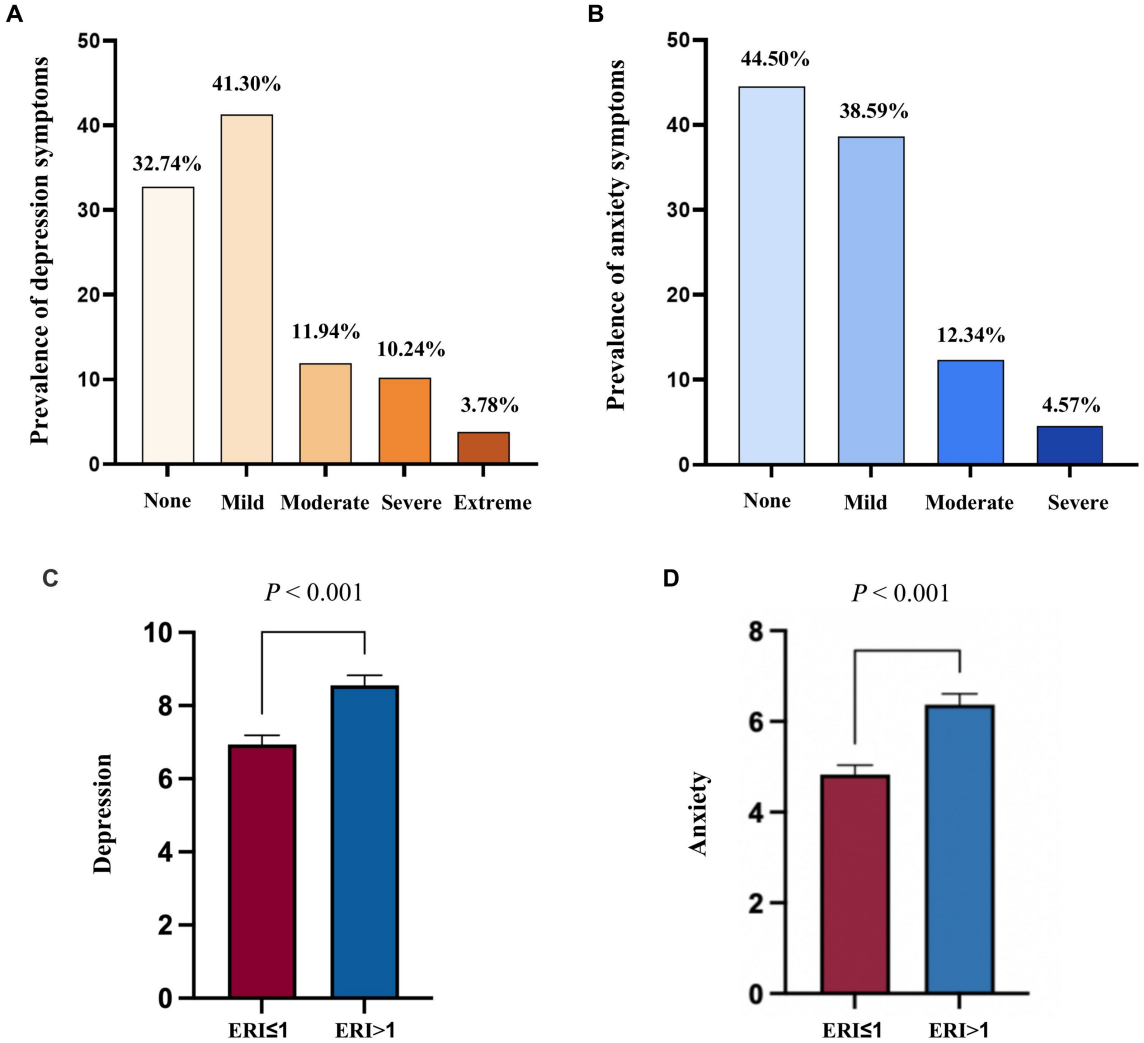
Distribution and differences in work stress and mental health problems among primary public health workers **(A)** Prevalence of depression symptom among primary public health workers; **(B)** Prevalence of anxiety symptom among primary public health workers; **(C)** Difference in depression symptom among primary public health workers with and without work stress; **(D)** Difference in anxiety symptom among primary public health workers with and without work stress; *p* calculated by t-test analysis.

Among the 3,809 PHWs, the number and percentage of public health physicians, general practitioners, nurses, administrative staff, medical technicians and other staff were 1,664 (43.69%), 298 (7.82%), 1,565 (41.09%), 32 (0.84%), 92 (2.41%) and 158 (4.15%), respectively. The prevalence of “no depression,” “mild depression,” “moderate depression,” “severe depression” and “extremely severe depression” among all participants were 32.74%, 41.30%, 11.94%, 10.24% and 3.78%, respectively. Furthermore, 44.50% of public health workers had no anxiety symptoms, while 55.50% had different degrees of anxiety symptoms, including 38.59% with “mild anxiety,” 12.34% with “moderate anxiety,” and 4.57% with “severe anxiety” (Figure 1). The differences of anxiety and depression symptom scores among participants across general characteristics were shown in Table 1. In addition, PHW with work stress had significantly higher anxiety (*t* = 9.46; *p* < 0.001) and depression scores (*t* = 8.53; *p* < 0.001) than those without work stress (Figure 1).

**Table 1:** Comparison of characteristics of anxiety and depression scores among public health workers (*N* = 3,809).

Characteristics	*N* (%)	Depression			Anxiety		
		Mean (SD)	t/F	*p*	Mean (SD)	t/F	*p*
Age (years)							
18–35	1729 (45.39)	8.04 (6.02)	21.69	<0.001	5.87 (5.20)	23.52	<0.001
36–45	1,333 (35.00)	7.96 (5.90)			5.77 (5.07)		
>45	747 (19.61)	6.43 (5.53)			4.42 (4.62)		
Gender							
Men	632 (16.59)	7.69 (6.42)	0.05	0.96	5.53 (5.33)	0.14	0.89
Women	3,177 (83.41)	7.70 (5.81)			5.56 (5.02)		
Educational level							
Junior college or below	986 (25.88)	7.60 (5.98)	0.27	0.77	5.45 (5.10)	1.172	0.31
Bachelor	2,743 (72.01)	7.73 (5.90)			5.57 (5.04)		
Master or above	80 (2.10)	7.98 (5.81)			6.34 (5.74)		
Marital status							
Single	575 (15.10)	8.29 (6.32)	3.48	0.03	6.07 (5.51)	3.52	0.03
Married or in a relationship	3,098 (81.33)	7.59 (5.83)			5.46 (4.97)		
Divorced or windowed	136 (3.57)	7.71 (6.09)			5.43 (5.28)		
Hometown type							
Large city	2010 (52.77)	8.05 (6.10)	8.30	<0.001	5.83 (5.23)	6.47	0.002
Medium-sized and small city	658 (17.27)	7.06 (5.53)			5.15 (4.88)		
Rural	1,141 (29.96)	7.45 (5.77)			5.30 (4.86)		
Type of occupation							
Public health physician	1,664 (43.69)	7.56 (5.88)	6.26	<0.001	5.53 (5.10)	5.86	<0.001
General practitioner	298 (7.82)	6.92 (5.55)			4.90 (4.77)		
Nurses	1,565 (41.09)	8.14 (6.04)			5.87 (5.15)		
Administrative staff	32 (0.84)	8.35 (6.27)			5.76 (4.86)		
Medical technicians	92 (2.41)	7.72 (5.43)			4.63 (4.05)		
Others	158 (4.15)	5.85 (5.15)			3.90 (4.44)		
Professional title grade							
None	387 (10.16)	7.29 (5.99)	2.25	0.08	5.03 (5.02)	3.16	0.02
Junior title	1,561 (40.98)	7.98 (6.03)			5.81 (5.21)		
Middle title	1770 (46.47)	7.56 (5.80)			5.46 (4.98)		
Vice-senior title or above	91 (2.39)	7.29 (5.90)			5.08 (4.44)		
Daily working time (hours)							
≤8	2,399 (62.98)	6.84 (5.41)	85.29	<0.001	4.81 (4.57)	88.53	<0.001
9–10	967 (25.39)	8.61 (6.16)			6.32 (5.45)		
>10	443 (11.63)	10.36 (6.87)			7.91 (5.80)		
Working years in the current institute (years)							
≤5	1,169 (30.69)	7.56 (5.78)	6.27	0.002	5.43 (5.04)	8.58	<0.001
6–15	1,536 (40.33)	8.09 (6.09)			5.94 (5.17)		
>15	1,104 (28.98)	7.30 (5.80)			5.14 (4.93)		
Length of public health service (years)							
≤5	1,354 (35.55)	7.94 (6.00)	5.45	0.004	5.73 (5.21)	5.06	0.006
6–15	1,677 (44.03)	7.78 (5.93)			5.65 (5.06)		
>15	778 (20.42)	7.09 (5.72)			5.04 (4.83)		
Work overtime							
Almost no overtime	395 (10.37)	5.21 (5.17)	129.38	<0.001	3.62 (4.43)	131.21	<0.001
Occasional overtime	2016 (52.93)	6.90 (5.24)			4.80 (4.48)		
Often overtime	1,398 (36.70)	9.55 (6.51)			7.18 (5.58)		
Cumulative time involved in front-line prevention during the COVID-19 pandemic (days)							
<40	373 (9.79)	6.53 (5.34)	432.62	<0.001	4.37 (4.36)	441.31	<0.001
40–79	428 (11.24)	7.13 (6.08)			4.99 (4.88)		
≧80	3,008 (78.97)	7.93 (5.95)			5.79 (5.16)		
Smoking							
No	3,621 (95.06)	7.68 (5.86)	0.74	0.46	5.54 (5.05)	0.42	0.68
Yes	188 (4.94)	8.06 (6.90)			5.71 (5.45)		
Drinking							
No	3,540 (92.94)	7.63 (5.87)	2.45	0.01	5.51 (5.03)	2.04	0.04
Yes	269 (7.06)	8.55 (6.50)			6.16 (5.55)		
Doing vigorous or moderate exercise							
No	1933 (50.75)	8.47 (6.12)	8.30	<0.001	6.20 (5.25)	8.09	<0.001
Yes	1876 (49.25)	6.90 (5.60)			4.88 (4.80)		
Daily sleep time (hours)							
<7	2041 (53.58)	9.41 (6.06)	20.40	<0.001	7.00 (5.30)	20.27	<0.001
≧7	1768 (46.42)	5.72 (5.08)			3.88 (4.22)		

### Correlation between Key variables

**Figure 2:**
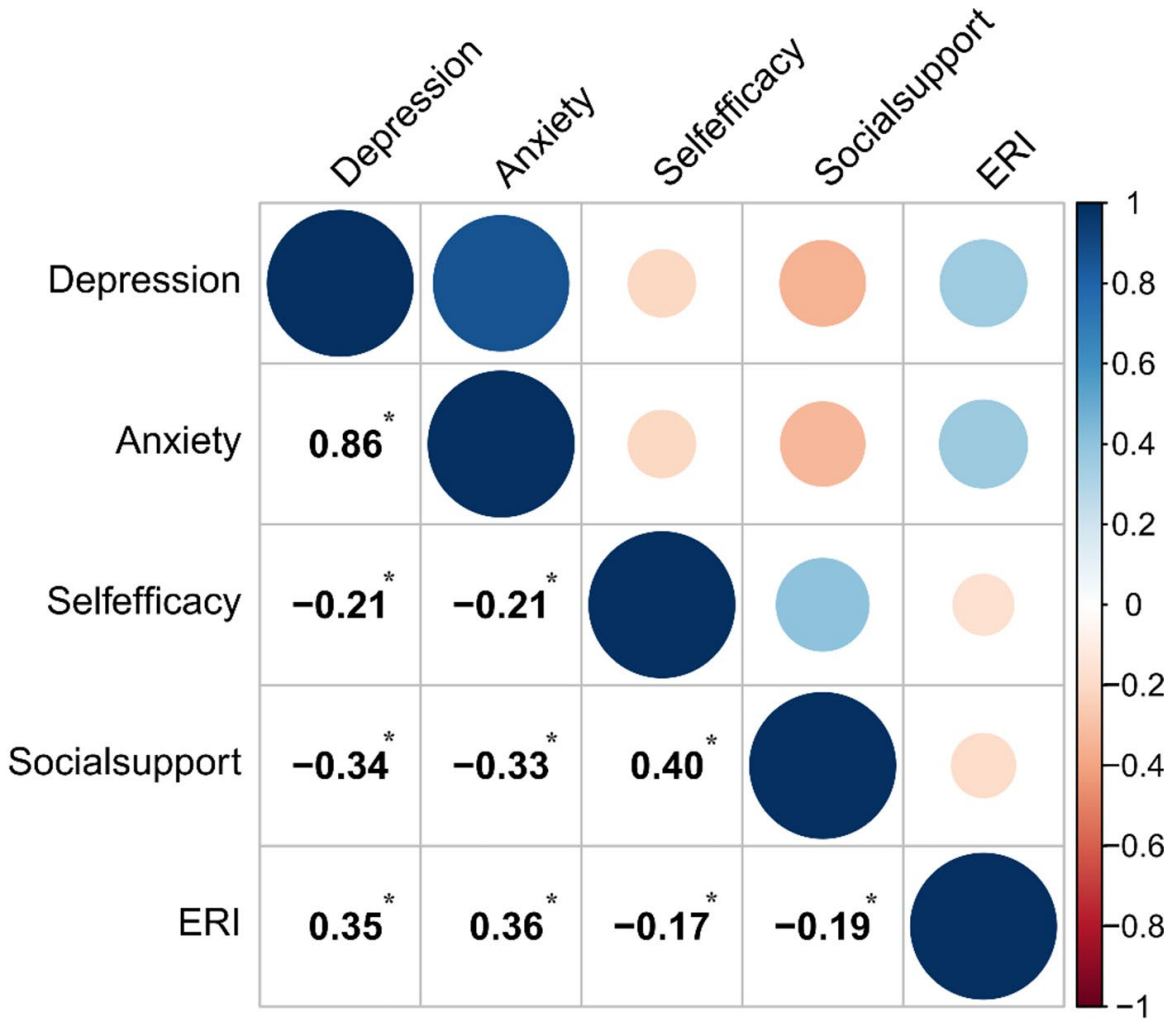
Correlations matrix plot of the main study variables. *p < 0.001.

Figure 2 presented the correlation matrix for key study variables. The Pearson correlation analysis showed that ERI was positively correlated with depression (*r* = 0.35, *p* < 0.001) and anxiety (*r* = 0.36, *p* < 0.001), but negatively related with social support (*r* = −0.19, *p* < 0.001) and self-efficacy (*r* = −0.17, *p* < 0.001). Depression was negatively and significant correlated with social support (*r* = −0.34, *p* < 0.001) and self-efficacy (*r* = −0.21, *p* < 0.001). This significantly negative correlations were also observed between anxiety and social support and self-efficacy (*p* < 0.001). The validity of Hypothesis 1 was confirmed by the results of the correlation analysis.

### Hierarchical multiple regression analysis

In the first step, demographic variables, lifestyle and COIVD-19-related and other work variables accounted for 17.0% and 17.1% of the variance in anxiety and depression symptoms, respectively (Table 2). In a second step, work stress was introduced into the model and was positively associated with anxiety and depressive symptoms, explaining 5.8% and 5.1% of the variance, respectively (standardized β=0.266, *p* < 0.001 and standardized β = 0.250, *p* < 0.001). The relationship between work stress and anxiety and depressive symptoms remained significant when social support and self-efficacy were finally added to the model, explaining 5.9% and 6.4% of the variance in anxiety and depression, respectively. The results of the above hierarchical multiple regressions also provided the basis and theoretical support for the complex relationships constructed by the structural equation model.

**Table 2:** The hierarchical linear regression analysis for mental health problems (*N* = 3,809).

Variables	Depression			Anxiety		
	Step 1 (β)	Step 2 (β)	Step 3 (β)	Step 1 (β)	Step 2 (β)	Step 3 (β)
Gender (Ref: female)	−0.016	−0.007	−0.027	−0.017	−0.008	−0.028
Age (Ref: 18-35)						
36–45	−0.015	−0.012	−0.012	−0.018	−0.014	−0.014
>45	−0.096^***^	−0.081^***^	−0.076^***^	−0.098^***^	−0.082^***^	−0.077^***^
Educational levels (Ref: junior college or below)						
Bachelor	−0.033^*^	−0.040^*^	−0.037^*^	−0.042^*^	−0.049^**^	0.046^**^
Master or above	−0.002	−0.001	−0.001	0.011	−0.010	−0.009
Marital status (Ref: single)						
Married or in a relationship	−0.051^**^	−0.047^**^	−0.039	−0.051^**^	−0.047^**^	−0.039^*^
Divorced or windowed	−0.018	−0.011	−0.010	−0.022	−0.015	−0.014
Hometown type (Ref: large city)						
Medium-sized and small city	−0.057^***^	−0.050^***^	−0.053^***^	−0.049^**^	−0.042^**^	−0.045^**^
Rural	−0.042^**^	−0.036^**^	−0.043^***^	−0.048^**^	−0.042^**^	−0.048^**^
Smoking (Ref: no)	0.003	0.005	0.003	0.002	0.001	−0.001
Drinking (Ref: no)	0.033^*^	0.027	0.026	0.028	0.022	0.020
Physical activity (Ref: no)	−0.101^***^	−0.089^***^	−0.065^***^	−0.097^***^	−0.085^***^	−0.061^***^
Sleep time (Ref: <7 h)	−0.251^***^	−0.215^***^	−0.188^***^	−0.249^***^	−0.211^***^	−0.185^***^
Type of occupation (Ref: others)						
Public health physician	0.032	0.021	0.026	0.065^*^	0.053	0.057^*^
General practitioner	0.021	0.020	0.022	0.032	0.031	0.033
Nurses	0.088^***^	0.081^**^	0.076^**^	0.101^***^	0.094^***^	0.090^***^
Professional title (Ref: None)						
Junior title	0.050	0.038	0.031	0.074^***^	0.061^**^	0.054^*^
Middle title	0.051	0.029	0.024	0.079^**^	0.057^*^	0.051^*^
Vice-senior title or above	0.012	0.005	0.007	0.015	0.008	0.009
Daily working time (Ref: ≤8)						
9–10	0.040^*^	0.019	0.010	0.036^*^	0.014	0.005
>10	0.073^***^	0.047^***^	0.051^***^	0.074^***^	0.047^**^	0.051^**^
Yeas of working in the current institute	0.041	0.032	0.024	0.023	0.013	0.006
Length of public health service	−0.041	−0.049^*^	−0.051^*^	−0.037	−0.045^*^	−0.047
Work overtime (Ref: almost no overtime)						
Often overtime	0.245^***^	0.156^***^	0.145^***^	0.220^***^	0.126^***^	0.115^***^
Occasional overtime	0.112^***^	0.063^*^	0.046^*^	0.080^**^	0.028	0.012
Cumulative time involved in front-line prevention during the COVID-19 pandemic in Shanghai (Ref: <40)						
40–79	0.046^*^	0.049^*^	0.044^*^	0.050^*^	0.053^**^	0.049^**^
≧80	0.051^*^	0.070^***^	0.045^*^	0.035^*^	0.062^**^	0.043^*^
Work stress (ERI)		0.250^***^	0.201^***^		0.266^***^	0.219^***^
Social support			−0.241^***^			−0.233^***^
Self-efficacy			−0.045^***^			−0.041^**^
*R* ^2^	0.171	0.222	0.286	0.170	0.228	0.287
Δ*R*^2^	0.171	0.051	0.064	0.170	0.058	0.059

*p < 0.05. **p < 0.01. ***p < 0.001. β coefficient is the standardized coefficient.

### Mediating effect analysis

**Figure 3:**
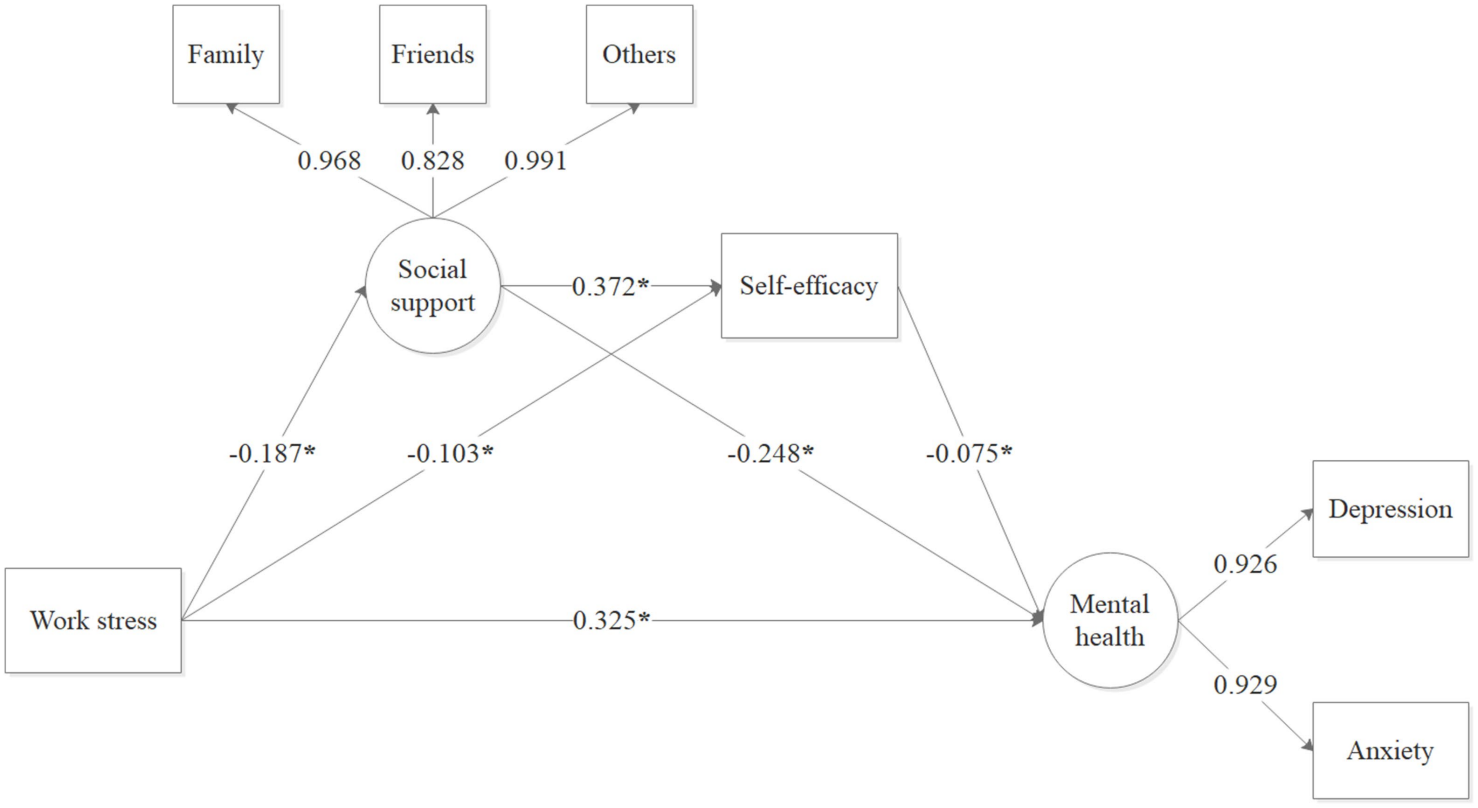
The mediating role of social support and self-efficacy in the relationship between work stress and mental health problems. *p < 0.001.

The results showed that five factors with eigenvalues greater than 1, and the first factor explained 32.40% of the variance (less than 40%). Therefore, it may be deduced that the variables involved in this study do not have significant common method bias. The results of mediation pathway model in the association between work stress and mental health were shown in Table 3 and Figure 3. All path coefficients, including direct and indirect effects, between work stress and mental health were statistically significant (*p* < 0.001, bias-corrected 95% confidence interval not including 0). The standardized direct effect of work stress on depression and anxiety was 0.325 (*p* < 0.001). The results indicated that work stress had a significant negative effect on social support (β = −0.187, *p* < 0.001) and self-efficacy (β = −0.103, *p* < 0.001). In addition, social support (β = −0.248, *p* < 0.001) and self-efficacy (β = −0.075, *p* < 0.001) had a significant negative influence on depression and anxiety. Notably, social support was positively associated with self-efficacy (β = 0.372, *p* < 0.001). The chain mediating effect model were acceptable according to the model fit index (model fit: χ2/df = 2.98 < 3 (*p* < 0.001), Tucker-Lewis index (TLI) = 0.982, comparative fit index (CFI) = 0.991, standardized root mean square residual (SRMR) = 0.026 and root mean square error of approximation (RMSEA) = 0.068 (*p* < 0.001)).

**Table 3:** Direct, indirect, and total effects of the chain mediation model between work stress and mental health problems.

Model pathways	Effect (standardized)	SE	95% CI	*p*
Total effect of X on Y	0.384	0.016	0.352–0.415	<0.001
Direct effect of X on Y	0.325	0.016	0.294–0.356	<0.001
Total Indirect effect of X on Y	0.059	0.007	0.046–0.074	<0.001
Indirect effect 1: X → M1 → Y	0.046	0.006	0.036–0.058	<0.001
Indirect effect 2: X → M2 → Y	0.008	0.003	0.003–0.014	0.004
Indirect effect 3: X → M1 → M2 → Y	0.005	0.002	0.002–0.009	0.001

## Discussion

Currently, there are limited epidemiological evidence on the association between work stress and mental health among primary public health workers. Overall, the prevalence of work stress and mental health problems (i.e., depression and anxiety) among community PHWs was 67.3 and 55.5%, respectively. The prevalence of mental health problems in our study was significantly higher than the combined prevalence of general healthcare workers reported in several meta-analyses during the COVID-19 pandemic (14, 41–43), which also emphasized the importance of focusing on the mental health for primary PHWs. Consistent with previous hypotheses, the current study showed that work stress had direct and indirect effects on depression and anxiety in primary PHWs. Furthermore, the mediating effect of social support and self-efficacy buffered the positive predictive effects of work stress on depression and anxiety. Exploring the underlying mechanisms and pathways between work stress and mental health, and identifying risk factors after a major public health event, is beneficial in preventing the occurrence and progression of mental health problems in primary PHWs.

As assumed, work stress was found to be directly associated with depression and anxiety among community PHWs, which supported and expanded the previous findings on occupational mental health among primary healthcare workers or medical staff. For example, a cross-sectional study conducted in Brazil found a significant positive relationship between ERI and common mental disorders among healthcare workers (44). Similar results were found for the significant positive effects of work stress on anxiety and depression among Chinese healthcare workers and medical staff (45, 46). Therefore, future research should pay more attention to the work and psychosocial situation of primary PHWs. Meanwhile, the related institutions are recommended to adopt positive coping strategies, such as improving job benefits, providing solid job security and continuous mental health services, to prevent and alleviate mental health problems caused by work stress (47).

As predicted, social support played a significant mediating role in the association between work stress and mental health among community PHWs, which explained 12.0% of the effect of work stress on anxiety and depression. This significant direct effect is consistent with the previous studies conducted with medical staff and primary healthcare workers (19, 45, 48). Individuals with higher levels of social support tended to experience professional achievement and increased confidence in coping with stressful situations, which contributed to reducing anxiety (49). Hence, nurses with higher levels of social support had positive emotional states (22, 50). On the contrary, medical staff who have lower levels of social support are at higher risks of developing depressive symptoms (51). Therefore, higher levels of social support may mitigate the negative effects of work stress on depression and anxiety.

In line with hypothesis, self-efficacy mediated the relationship between work stress and mental health among PHWs. This means that work stress can reduce PHWs’ self-efficacy, which in turn can lead to depression and anxiety. Individuals with higher self-efficacy have greater confidence in their ability to handle and overcome work-related challenges and more likely to adopt positive coping strategies to achieve successful and satisfying performance, which may explain why self-efficacy can buffer the effects of work-related stress on mental health (32, 52). Thus, high occupational stress among hospital sanitation workers leads to reduce self-efficacy, which in turn puts them at higher risk of suffering from poor mental health during the COVID-19 epidemic (21). The current study conducted in primary PHWs fulfilled and expanded inadequate research findings on self-efficacy, while highlighting the need for interventions that focus on enhancing self-efficacy to alleviate the adverse effects of job stress on mental health.

Notably, the chain mediation role of social support and self-efficacy in the association between work stress and mental health (depression and anxiety) was first demonstrated in primary PHWs. The long-term accumulation of work-related stress can lead to lack of social support from family, friends and others due to busy work, and consequently losing self-confidence to cope with setbacks and difficulties, eventually leading to a significant increase in anxiety and depression (22). Moreover, social support, as a protective factor for self-efficacy, can provide additional external resources to help improve self-efficacy when individuals perceive work stress (23). And individuals with higher self-efficacy in the process of connecting with people who provide social support may positively cope with work stress and effectively reduce the adverse effects of work stress on mental health (53). Therefore, the current study showed the sequential mediating effect of social support and self-efficacy in the relationship between work stress and mental health, which also provides a potential mechanism for the interaction between individual internal characteristics (e.g., self-efficacy) and external environment (e.g., social support). The indirect effect sizes of social support and self-efficacy on work stress and mental health were not high, respectively, although such indirect effects were shown to be significant. This might be related to factors associated with blocking social support such as lockdowns and social isolation during COVID-19. The relationship and pathways between work stress and mental health are complex, which in turn indicates the need to explore other pathways and potential mechanisms between work stress and mental health. Finally, based on these, related institutions can implement more targeted psychological interventions to improve the psychological health and service quality of community PHWs.

Previous literature suggested that some psychological interventions might reduce anxiety and depressive symptoms by increasing self-efficacy and accessing more social support. For example, effective coping strategies, such as setting up special rest areas and counseling rooms, providing individual case work and group work, using online social platforms and developing healthy and optimistic behaviors, could obtain adequate social support from within or outside the family for coping with work stress and challenges, and reducing anxiety and depression (48). Group-based activities and psychiatric training that improved self-efficacy could reduce depression and anxiety symptoms (32, 53). In addition, interventions that simultaneously consider the interactive effects of social support and self-efficacy may be equally important in reducing the risk of anxiety and depression.

Several limitations of the current study should be considered. First, we failed to examine the causal relationships between work stress, social support, self-efficacy, and mental health due to the limitation of cross-sectional design. Therefore, the intrinsic mechanisms between variables should be further explored in more detail, combined with experimental and long-term longitudinal studies. Second, the main variables in this study were assessed by self-rating scales, which may not avoid resulting recall and social desirability bias. Third, primary PHWs information in this study was collected from all community health centers, but only from one city in China. Therefore, future studies need to conduct surveys in more cities to improve the generalizability of our findings.

Despite the above limitations of this study, this large-scale cross-sectional study with large sample sizes extended the literature on the direct and indirect relationship between work stress and mental health among primary PHWs. The findings of this study shed light on the critical role of social support and self-efficacy in alleviating work stress-induced anxiety and depressive symptoms among community PHWs. The present study contributed to explore the chain mediating pathways of social support and self-efficacy between work stress and mental health, which finding provides a new perspective on interventions to address work stress and mental health among primary PHWs. Therefore, this study has significant public health implications and provides a theoretical and practical basis for government and managers of primary health service center institutions to adopt targeted interventions.

## Conclusion

This study showed a high prevalence of depression and anxiety symptoms among primary public health workers after the COVID-19 pandemic outbreak. Moreover, this study revealed that work stress has a significant positive direct effect on depression and anxiety symptoms among PHWs, with social support and self-efficacy playing an independent mediating role. These findings may provide a theoretical basis for developing psychosocial interventions for mental health of primary PHWs in China. Therefore, related healthcare institutions should pay more attention to the mental health of primary public workers, and enhancing social support for public health workers and improving their self-efficacy may be an effective approach to alleviate mental health in follow-up interventions.

## Data availability statement

The raw data supporting the conclusions of this article will be made available by the corresponding authors upon reasonable request.

## Ethics statement

The studies involving human participants were reviewed and approved by the Ethical Review Committee of School of Public Health, Shanghai Jiao Tong University School of Medicine (approval number: SJUPN-202108) and adhered to the principles of the Declaration of Helsinki. The patients/participants provided their written informed consent to participate in this study. Written informed consent was obtained from the individual(s) for the publication of any potentially identifiable images or data included in this article.

## Author contributions

YD: conceptualization, investigation, data curation, formal analysis, and writing – original draft. QZ: investigation, methodology, formal analysis, validation, and writing – original draft. RC: investigation, validation, and data curation. RW: investigation, methodology, and data curation. HH: conceptualization, supervision, and writing – review & editing. YC: conceptualization, funding acquisition, supervision, and writing review & editing. All authors contributed to the article and approved the submitted version.

## Funding

This study was supported National Key R&D Program of China, Strategic Collaborative Innovation Team (2018YFC1705103). Shanghai Three-Year Action Plan for Public Health under Grant (GWV-10.1-XK18 and GWV-10.2-XD13). Shanghai Municipal Education Commission (2023-PHAPD-01-45). Hospital Management Program, China hospital development institute, Shanghai Jiao Tong University (CHDI-2022-B-05). Science and Technology Commission Shanghai Municipality (No. 20JC1410204).

## Conflict of interest

The authors declare that the research was conducted in the absence of any commercial or financial relationships that could be construed as a potential conflict of interest.

## Publisher’s note

All claims expressed in this article are solely those of the authors and do not necessarily represent those of their affiliated organizations, or those of the publisher, the editors and the reviewers. Any product that may be evaluated in this article, or claim that may be made by its manufacturer, is not guaranteed or endorsed by the publisher.
