# Supplementary figures and images for: Association between work stress and mental health in Chinese public health workers during the COVID-19 epidemic: mediating role of social support and self-efficacy
Source: Front Public Health. 2023 Jul 27;11:1236645. doi: 10.3389/fpubh.2023.1236645 (PMC10415911; doi:10.3389/fpubh.2023.1236645)

**Supplementary**


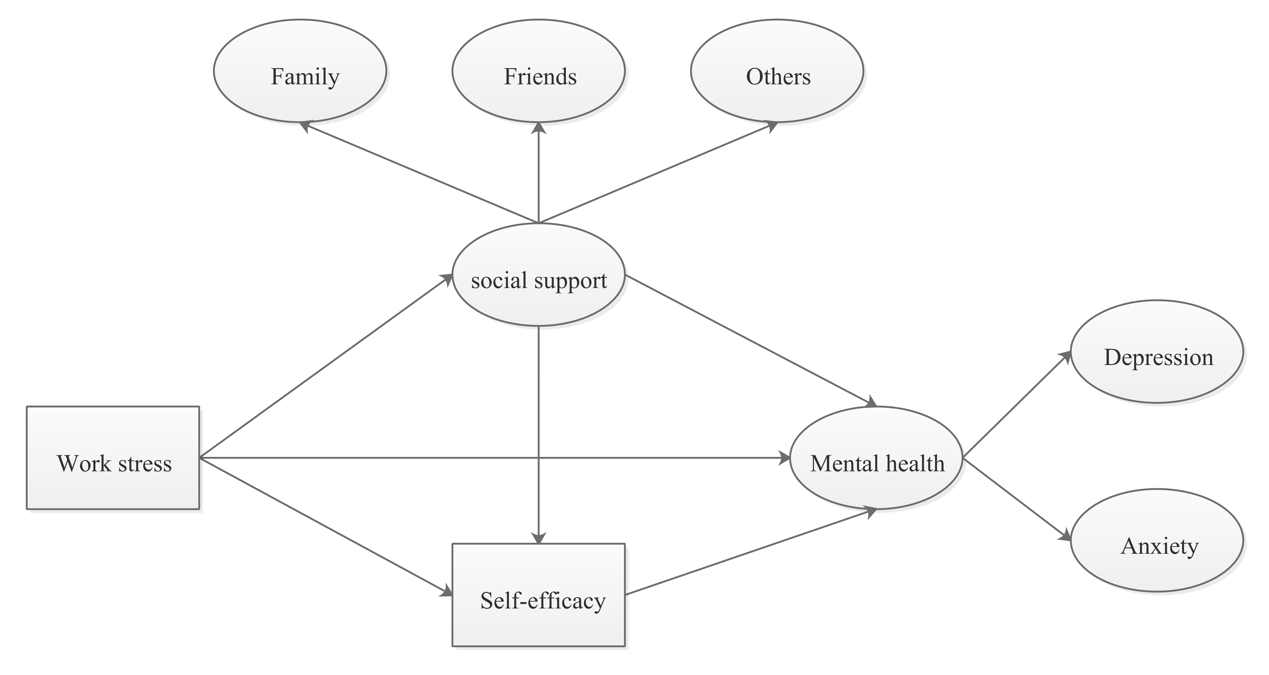


**Figure 1.** Hypothetical chain mediation model

Supplement: Supplementary file 1 [file Data_Sheet_1.docx]
